# Catheter-directed mechanical thrombectomy in a patient with high-risk pulmonary embolism complicated by out-of-hospital cardiac arrest: a case report

**DOI:** 10.1093/ehjcr/ytad307

**Published:** 2023-07-12

**Authors:** Shifan Thangavel, Kasper Korsholm, Karsten Tange Veien, Kim M Larsen, Asger Andersen

**Affiliations:** Department of Cardiology, Aarhus University Hospital, Palle Juul-Jensens Boulevard 99, 8200, Aarhus N, Denmark; Department of Cardiology, Aarhus University Hospital, Palle Juul-Jensens Boulevard 99, 8200, Aarhus N, Denmark; Department of Cardiology, Aarhus University Hospital, Palle Juul-Jensens Boulevard 99, 8200, Aarhus N, Denmark; Prehospital Emergency Medical Services, Central Denmark Region, Aarhus N, Denmark; Department of Cardiology, Aarhus University Hospital, Palle Juul-Jensens Boulevard 99, 8200, Aarhus N, Denmark

**Keywords:** Pulmonary embolism, Catheter-directed treatment, PERT, Case report

## Abstract

**Background:**

Pulmonary embolism (PE) is common, and it is the third leading cause of cardiovascular death. The management of patients with high-risk PE generally consists of systemic thrombolysis; however, surgical or catheter-directed treatment (CDT) can be considered in selected cases.

**Case summary:**

A 78-year-old female patient presenting with acute severe dyspnoea develops out-of-hospital cardiac arrest (OHCA). She was admitted with return of spontaneous circulation and a critical haemodynamic state upon arrival to the catheterization laboratory with an estimated no-flow time of 1 min and low-flow time of 52 min. An acute pulmonary angiogram reveals massive PE. After a PE response team conference, the patient was not found eligible for extracorporeal membrane oxygenation, surgery, or thrombolysis. The patient was treated with catheter-directed mechanical thrombectomy 129 min after first medical contact. The patient recovered and was discharged without any neurological deficits.

**Discussion:**

Catheter-directed mechanical thrombectomy was a successful treatment in a patient with OHCA secondary to high-risk PE, where thrombolysis and surgical interventions were considered contraindicated. This case underlines the future perspectives of CDT and also that a multidisciplinary team approach may benefit patients with high-risk PE.

**Learning points**

- A multidisciplinary team approach may benefit patients with cardiac arrest caused by pulmonary embolism (PE) for the best treatment of choice.
- Catheter-directed mechanical embolectomy could be a feasible alternative to thrombolysis or surgery in patients with cardiac arrest caused by PE.

## Introduction

Pulmonary embolism (PE) is common, with incidence rates reported in different countries ranging from 39 to 115 per 100 000 per year, and it is the third leading cause of cardiovascular death.^1^ The clinical presentation of PE may vary from asymptomatic to cardiac arrest, and management can be challenging due to comorbidities and PE complications.^2^ The pulmonary obstruction caused by the clot leads to an increase in right ventricular afterload and a risk of right ventricular failure with subsequent haemodynamic collapse.^2^ The management of patients with high-risk PE generally consists of systemic thrombolysis; however, surgical or catheter-directed treatment (CDT) may be considered in select cases.^3^ Treatment with systemic thrombolysis is effective but carries a substantial risk of bleeding. Hence, its recommendation is currently restricted to haemodynamically unstable patients with an immediate need to restore pulmonary perfusion classified as high risk in the 2019 ESC guidelines.^2^ Catheter-directed treatment is an evolving field covering catheter-directed thrombolysis or mechanical thrombectomy. These therapies may prove beneficial in restoring pulmonary perfusion while reducing the risk of bleeding or adverse events compared with thrombolysis or surgical interventions.^3,4^

In this case, we describe a 78-year-old female who suffered out-of-hospital cardiac arrest (OHCA) caused by PE. She was successfully treated with catheter-directed mechanical thrombectomy and achieved full neurological recovery.

## Summary figure

**Table:** 

Day −19	Total left knee replacement. Discharged with compression stockings.
Day −1	Debut of dyspnoea.
Day 0 Time 0	Cardiac arrest. Advanced life support was initiated. Intermittent return of spontaneous circulation and cardiac arrest during transport.
Time +88 min	Arrival at catheterization laboratory with spontaneous circulation on high-dose vasopressor. No-flow time 1 min. Low-flow time 52 min. Echocardiography with severe dilatation of the right ventricle.
Time +113 min	Pulmonary angiogram with bilateral central pulmonary embolism (PE).
Time +129 min	PE response team call: Patient was considered ineligible for extracorporeal membrane oxygenation due to age and comorbidities. Catheter-directed mechanical thrombectomy was considered the best and safest option and was performed immediately with aspiration of large clot masses and normalization of haemodynamics.
Time +156 min	Transfer to intensive care unit.
Day 1	Patient awakens with relevant contact and with normal haemodynamics. Patient is extubated.
Day 2	Unfractionated heparin was changed to low-molecular-weight heparin.
Day 5	Transfer from intensive ward to cardiology ward.
Day 16	Transfer to local hospital.
Day 27	Discharged from local hospital with lifelong non-vitamin K antagonist oral anticoagulant.

## Case presentation

A 78-year-old female’s medical history included hypertension and transient cerebral ischaemia. Former cardiac manifestations included Takotsubo cardiomyopathy based on acute heart failure with ejection fraction (EF) of 35%, a normal coronary angiogram, a cardiac magnetic resonance imaging (MRI) scan, and a full recovery of left ventricular EF.

**Figure 1:**
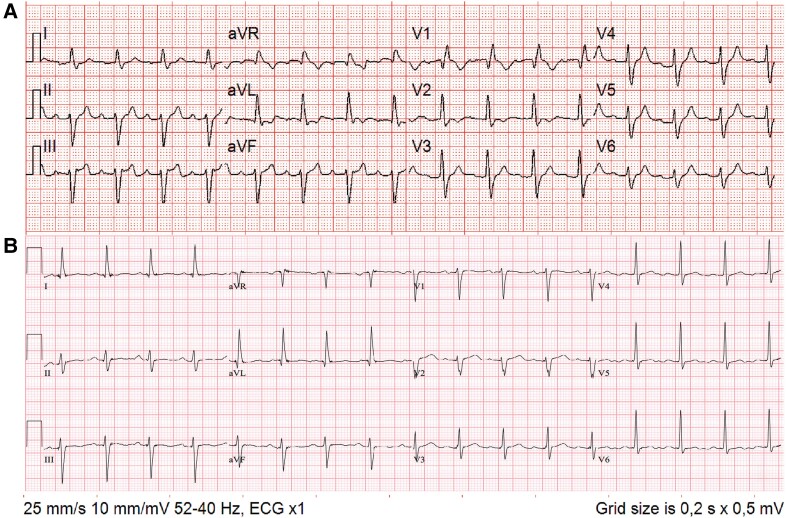
(*A*) Electrocardiogram obtained pre-hospital showing sinus rhythm with right bundle branch block with ST-segment elevation in inferior leads. (*B*) Electrocardiogram after pulmonary thrombectomy showing sinus rhythm without right bundle branch block and ST-segment elevation.

The patient underwent left knee replacement surgery with brief transient immobilization without prophylactic anticoagulation. On Day 18 after surgery, the patient suddenly developed dyspnoea and contacted the emergency medical service (EMS) 12 h after the onset of symptoms. Upon EMS arrival, the patient collapsed with cardiac arrest and pulseless electrical activity (PEA) at first rhythm control. Pre-hospital cardiopulmonary resuscitation (CPR) was immediately provided according to the European Resuscitation Council (ERC) 2021 guidelines for advanced life support.^5^ At return of spontaneous circulation (ROSC), a pre-hospital electrocardiogram (ECG) revealed a new right bundle branch block (RBBB) with ST-segment elevation in inferior leads (*Figure 1*). The patient was intubated, and mechanical respiratory support was initiated; ROSC was achieved twice, but the patient deteriorated and was transferred to the catheterization laboratory (cathlab) with ongoing mechanical CPR using the Lund University Cardiac Assist System (LUCAS). Repeated doses of adrenaline were administered during CPR, totalling 6 mg. Upon arrival at the cathlab, the patient had ROSC at rhythm analysis and an end-tidal carbon dioxide level of 3.4 kPa. Estimated no-flow time was 1 min and low-flow time was 52 min.

**Figure 2:**
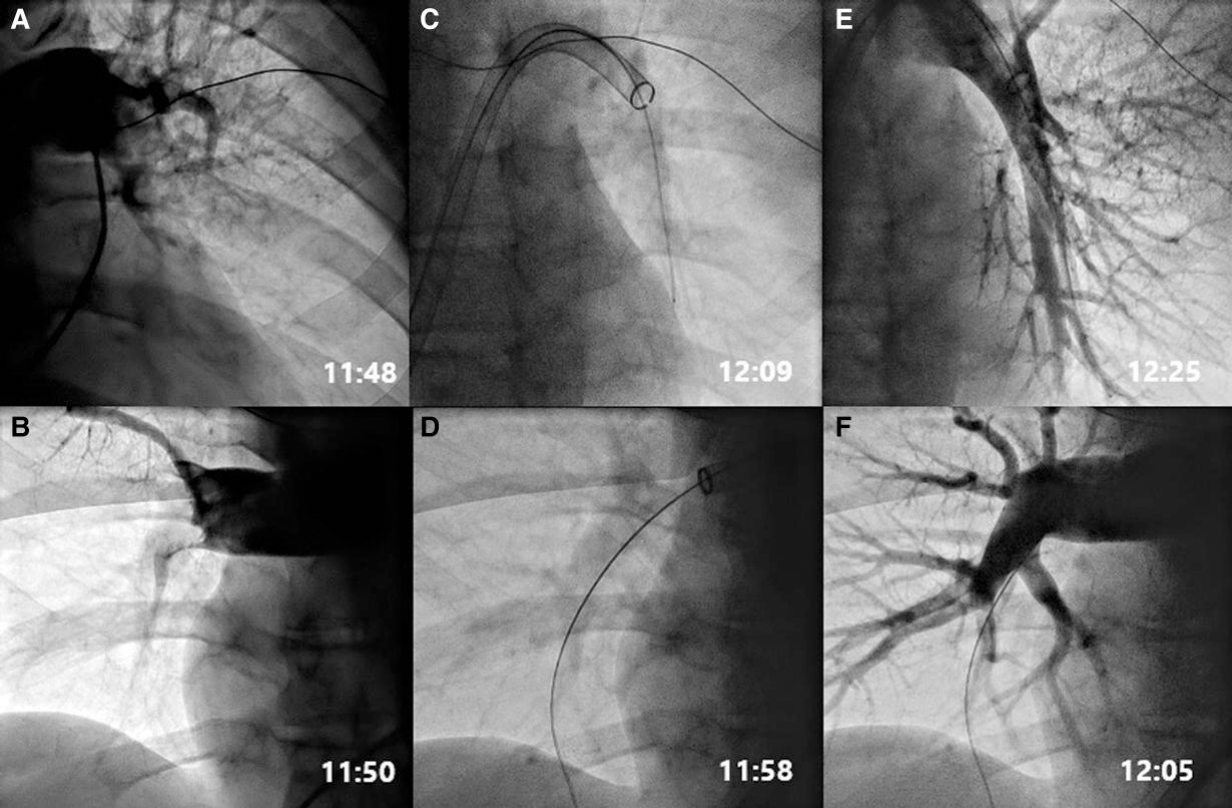
(*A*) Left pulmonary artery with embolus. (*B*) Right pulmonary artery with embolus. (*C*) Aspiration catheter placed in the left pulmonary artery. (*D*) Aspiration catheter placed in the right pulmonary artery. (*E*) Left pulmonary artery after thrombectomy. (*F*) Right pulmonary artery after thrombectomy.

Echocardiography at arrival in the cathlab revealed severe dilatation of the right ventricle. The first arterial blood gas showed pH at 6.59 (7.37–7.45), lactate 14.9 mmol/L (0.5–2.5), glucose 19.7 mmol/L (4.2–7.8), and potassium 5.7 mmol/L (3.5–4.6). The most likely reversible cause of the cardiac arrest was PE, and an immediate pulmonary angiogram confirmed obstruction of the main pulmonary arteries (*Figure 2*). Continuous norepinephrine infusion, 0.20 μg/kg/min, was required to maintain an invasive mean arterial pressure of ∼60 mmHg.

The management was discussed with the PE response team (PERT).^6^ Based on the patient’s age, prolonged low-flow time, and comorbidities, the patient was deemed ineligible for extracorporeal membrane oxygenation (ECMO) and surgical thrombectomy. Systemic thrombolysis was considered high risk due to gender, age, prolonged mechanical CPR, and recent surgery.^4^ Nevertheless, immediate reperfusion was urgently required to restore haemodynamics, and catheter-directed mechanical thrombectomy was considered the best treatment of choice.

**Figure 3:**
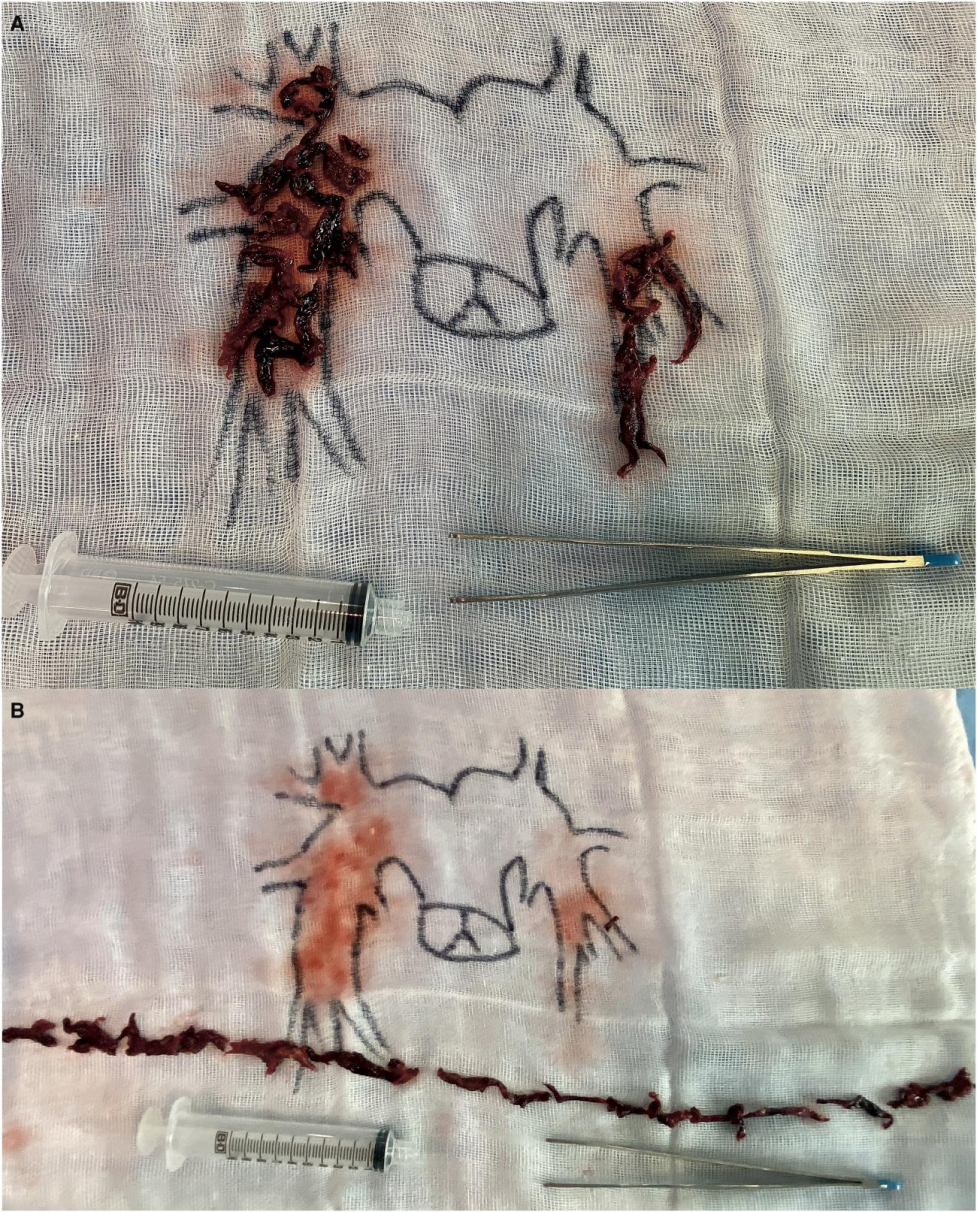
(*A*, *B*) Aspirated clot masses.

The thrombus aspiration was performed through right femoral vein access using the 24-Fr Inari FlowTriever Aspiration Catheter (Inari Medical, Irvine, CA, USA). Large clot masses were successfully aspirated, restoring pulmonary perfusion and haemodynamics (*Figure 3*). The time from the first contact with EMS to pulmonary reperfusion was 2 h and 31 min, and the thrombus aspiration procedure time was 20 min.

After the procedure, the patient was admitted to the intensive care unit (ICU). Head, thoracic, abdominal, and pelvic computed tomography (CT) scans showed some residual bilateral PE from the hilus region and peripherally and several fractured ribs. Anticoagulation with unfractionated heparin and target activated partial thromboplastin time (APTT) (20–29) between 45 and 60 was administered the first 2 days after the procedure, and thereafter, low-molecular-weight heparin (LMWH) was initiated. The patient was extubated on the first post-procedural day without serious neurological deficits and discharged from the ICU to the cardiology ward after 5 days. She fully recovered from her PE, but admission was prolonged due to gastrointestinal *Clostridium difficile* infection. After 27 days, she was discharged home with apixaban 5 mg × 2.

## Discussion

Patients presenting with PE and cardiac arrest can pose a challenging clinical problem. Patients presenting with cardiac arrest should be managed according to the ERC guidelines,^5^ which include evaluation and management of possible reversible causes. Anamnestic information was critical in the present case, but ECG and ultrasound can be early diagnostic tools to establish a tentative diagnosis and guide initial treatment. Despite trials showing no benefit of early invasive coronary angiography in OHCA cases without ST-elevations on ECG,^7^ the cathlab may be an optimal place to admit patients with haemodynamic collapse or OHCA suspected to have PE, as several advanced diagnostics and treatments are readily available. Guidelines recommend a CT pulmonary angiogram (CTPA) as the first-choice diagnostic imaging modality in suspected PE.^8^ However, CTPA may not be readily available and should not delay vital treatment. The suspicion of PE should ideally be confirmed before initiation of reperfusion therapy, as right ventricular dilatation during cardiac arrest may have other causes than PE. An alternative to CTPA is a pulmonary angiogram^2^ that can be performed without considerable time delay in the cathlab, and immediate treatment including ECMO, CDT, or systemic thrombolysis will be readily available.

According to the 2019 ESC PE guidelines, systemic thrombolysis remains the first-line treatment in high-risk PE.^2^ Absolute or relative contraindications for thrombolysis require consideration of other treatment strategies like ECMO with surgical thrombectomy or CDT.^2^ Extracorporeal membrane oxygenation is not recommended as a stand-alone treatment, and evidence for its use in PE is sparse.^9^

Catheter-directed treatment is an evolving therapy with limited availability, but as illustrated in this case, patients with prolonged mechanical CPR due to cardiac arrest will be at high risk for bleeding, and CDT may be a good treatment option. The evidence behind CDT is primarily based on observational data^10^ and non-randomized trials^11^ comparing outcomes with systemic thrombolysis or anticoagulation strategies. The lack of randomized trials in CDT limits its current use, but as suggested in a recent ESC consensus statement, CDT should be considered in patients with OHCA caused by PE, high-risk PE, or intermediate-high-risk PE who are not improving on anticoagulation and with contraindication for systemic thrombolysis or failed thrombolysis.

We have not been able to identify any other cases in the literature, which describe successful CDT in patients with OHCA without the use of ECMO or systemic thrombolysis, why this case may be the first to describe such a patient history.

This case illustrates the importance of careful pre-hospital triage management, a multidisciplinary PERT set-up, and treatment of patients with OHCA, and it underlines that CDT may have promising future perspectives, but randomized trials are needed to support its use in PE patients.

## Conclusion

Catheter-directed mechanical thrombectomy was a successful treatment in a patient with OHCA caused by PE, where thrombolysis and surgical interventions were considered contraindicated.

## Data Availability

No new data were generated or analysed in support of this research.

## Lead author biography

Shifan Thangavel has been a medical doctor since 2013 and a cardiologist since 2019 and is working as a senior registrar at Aarhus University Hospital full time.

**Consent:** The patient has provided written informed consent for the publication of this case report.

**Funding:** None declared.
